# Photosystem II antenna modules CP43 and CP47 do not form a stable ‘no reaction centre complex’ in the cyanobacterium *Synechocystis* sp. PCC 6803

**DOI:** 10.1007/s11120-022-00896-w

**Published:** 2022-01-11

**Authors:** Martina Bečková, Roman Sobotka, Josef Komenda

**Affiliations:** Laboratory of Photosynthesis, Institute of Microbiology of the Czech Academy of Sciences, Centre Algatech, Opatovický mlýn, 37981 Třeboň, Czech Republic

**Keywords:** Photosystem II, Photosynthesis, CP43, CP47, No reaction centre complex

## Abstract

The repair of photosystem II is a key mechanism that keeps the light reactions of oxygenic photosynthesis functional. During this process, the PSII central subunit D1 is replaced with a newly synthesized copy while the neighbouring CP43 antenna with adjacent small subunits (CP43 module) is transiently detached. When the D2 protein is also damaged, it is degraded together with D1 leaving both the CP43 module and the second PSII antenna module CP47 unassembled. In the cyanobacterium *Synechocystis* sp. PCC 6803, the released CP43 and CP47 modules have been recently suggested to form a so-called no reaction centre complex (NRC). However, the data supporting the presence of NRC can also be interpreted as a co-migration of CP43 and CP47 modules during electrophoresis and ultracentrifugation without forming a mutual complex. To address the existence of NRC, we analysed *Synechocystis* PSII mutants accumulating one or both unassembled antenna modules as well as *Synechocystis* wild-type cells stressed with high light. The obtained results were not compatible with the existence of a stable NRC since each unassembled module was present as a separate protein complex with a mutually similar electrophoretic mobility regardless of the presence of the second module. The non-existence of NRC was further supported by isolation of the His-tagged CP43 and CP47 modules from strains lacking either D1 or D2 and their migration patterns on native gels.

**Supplementary Information:**

The online version contains supplementary material available at 10.1007/s11120-022-00896-w.

## Introduction

The photosystem II complex (PSII) is one of two photosynthetic reaction centre (RC) complexes performing photochemical energy conversion within oxygenic phototrophs. Compared with photosystem I (PSI), PSII performs more oxidative photochemistry by oxidizing water into dioxygen and such is more susceptible to photodamage (Barber 1995). The main mechanism which counteracts PSII photodamage is the PSII repair cycle, which is based on a selective replacement of the central PSII protein D1 (Silva et al. 2003; Komenda et al. 2006). The PSII repair is initiated by detachment of the CP43 antenna (Barbato et al. 1992; Komenda and Masojídek 1995) with bound small proteins and pigments, altogether called the CP43 module (CP43m, Boehm et al. 2011; Komenda et al. 2012b). Only in the absence of adjacent CP43 can the damaged D1 subunit be accessed and degraded by a membrane-bound FtsH protease complex. On the other hand, the degradation of D1 may occur even without photodamage if CP43 is missing or its binding is destabilized (Krynická et al. 2015). After insertion of the new D1 copy, the PSII complex is reassembled and oxygen evolution reactivated.

This fast PSII repair mechanism cannot be, however, utilized if the newly synthesized D1 is not immediately available or if the homologous D2 protein, which together with D1 binds cofactors performing the charge separation, is also damaged. Both D1 and D2 then must be degraded and the CP43m and the entire module of the second PSII antenna CP47 (CP47m) are released (Boehm et al. 2011; Komenda and Masojídek 1995). A recent study (Weisz et al. 2019) proposed that, in the cyanobacterium *Synechocystis sp.* PCC 6803 (hereafter *Synechocystis*), the antenna modules released from degraded PSII form a mutual complex that was named 'no reaction centre complex’ (NRC). NRC was postulated as another component of a wider set of transient complexes formed during the PSII repair. However, an alternative explanation of the results is that the free CP47m and CP43m have similar mobility during gel electrophoresis and glycerol gradient centrifugation and that the postulated complex is simply a mixture of the two free modules. Here, we studied mobility of CP43m and CP47m in high light-treated WT and several PSII mutants lacking either CP43 or CP47 antennae or lacking D2 and consequently accumulating both antenna modules together. In addition, we isolated each module from strains lacking either D1 or D2 and compared their mobility in different native gel systems. Our data demonstrate that the mobility of each module was independent of the presence or absence of the second module, which argues strongly against the formation of NRC.

## Materials and methods

### Strains and cultivation conditions

We used the glucose-tolerant GT-P wild-type strain of *Synechocystis*, hereafter denoted as WT (Tichý et al. 2016) and the following PSII-less strains derived from this WT: ∆*psbB* strain, in which the *psbB* gene encoding CP47 was replaced by spectinomycin resistance cassette (Eaton Rye and Vermaas 1991); ∆*psbC* strain, in which the *psbC* gene encoding CP43 was replaced by kanamycin resistance cassette (Vermaas et al. 1988); and ∆*psbE* strain, in which the whole *psbEFLJ* operon was replaced by chloramphenicol resistance cassette (Pakrasi et al. 1988). In addition, we used the *his*-*psaF* strain expressing 6xHis-tagged PsaF subunit of PSI (Kubota et al. 2010), and *his-psbC*/Δ*psbA* (Boehm et al. 2011) and *his-psbH/*Δ*psbH/*Δ*psbE/*Δ*ftsH2* strains (D’Haene et al. 2015) for the purification of PSI and individual ‘free’ CP43 and CP47 assembly modules, respectively.

WT and PSII mutants were cultivated in 250-ml flasks in liquid BG11 medium supplemented with 5 mM glucose, placed on an orbital shaker in temperature-controlled room (27–28 °C). Cells were illuminated with fluorescent white light of 40 μmol photons m^2−^ s^1−^ (normal light, NL) or 300 μmol photons m^2−^ s^1−^ (high light, HL). To accelerate photodamage of PSII, the cultures were transferred to an incubator set to 18 °C (low-temperature (LT) experiments). For the purification of His-tagged PsaF and His-CP43, the *his-psaF* and *his-psbC*/Δ*psbA* strains were cultivated in 4-L flasks in the presence of 5 mM glucose under NL, constant air bubbling and stirring. Light-sensitive *his-psbH/*Δ*psbH/*Δ*psbE/*Δ*ftsH2* cells were grown under the same conditions but at 10 μmol of photons s^−1^ m^−2^.

### Preparation of cellular membranes

Cells in exponential growth phase (30 ml, OD at 750 nm of 0.5) were pelleted, washed and resuspended in buffer A (25 mM MES/NaOH, pH 6.5, 10 mM CaCl_2_, 10 mM MgCl_2_ and 25% (w/v) glycerol). Cells were broken mechanically by vortexing with zirconia/silica beads (0.1 mm in diameter, BioSpec) in four cycles of one minute breaking and five minutes cooling on ice. The membrane and soluble fractions were separated by centrifugation (Sigma 3K30; 65,000 g, 20 min). The pelleted membranes were resuspended in buffer A.

### Purification and analysis of His-tagged PSI and PSII antenna modules

Purification of His-tagged PsaF, CP43m and CP47m in buffer A was performed as described in (Komenda et al. 2012a). His-tagged CP43m and CP47m were also isolated in buffer B (20 mM Hepes buffer pH 7.5, 5 mM CaCl_2_, 5 mM MgCl_2_ 10% glycerol). Membranes were then solubilized with 1% n-β-dodecyl-maltoside (w/v) at chlorophyll concentration of 1 mg/mL and the His-tagged PSI and CP47 and CP43 assembly modules were purified using nickel affinity chromatography (Protino Ni–NTA Agarose, Macherey–Nagel, Germany) essentially as described in (Shukla et al. 2018).

### Protein electrophoresis and immunoblotting

For native electrophoresis, membranes were solubilized with 1% n-β-dodecyl-maltoside while isolated complexes and modules were loaded directly without any other detergent addition. The solubilized membrane proteins, isolated in buffer A, were separated on 4–14% (w/v) polyacrylamide clear native (CN) gels (Wittig et al. 2007) while those isolated in buffer B were separated on 4–16% NativePAGE gel (ThermoFisher) in the first dimension. Markers used for NativePAGE electrophoresis were high molecular weight calibration kit (Amersham Biosciences) and NativeMARK unstained protein standard (ThermoFisher). The protein gels were scanned and the chlorophyll fluorescence image was taken by a LAS-4000 camera (Fuji). For 2D analysis, the cut individual lanes of the CN gel were incubated in solubilizing buffer (25 mM Tris/HCl, pH 7.5, 1% SDS and 1% dithiothreitol) for 30 min at room temperature with agitation and subsequently loaded on SDS PAGE (16–20% (w/v) polyacrylamide gel containing 7 M urea) for separation of the protein complexes in the second dimension. Proteins were stained by SYPRO Orange (Sigma-Aldrich) and photographed using a LAS-4000 camera (Fuji). Subsequently, the proteins from the gel were blotted to PVDF membrane (pore size of 40 μm), using wet transfer. Blot was blocked with 0.2% Tween, incubated with primary antibodies specific for CP47 (Agrisera, cat. no. AS04 038) and CP43 (Agrisera, cat. no, AS11 1787) and then with secondary antibody conjugated with horseradish peroxidase (Sigma-Aldrich). The blot was visualized using the chemiluminescent substrate Immobilon Crescendo (Millipore) and the signal was recorded using a LAS-4000 camera (Fuji).

## Results

### The unassembled CP47 and CP43 antenna modules have a very similar electrophoretic mobilities regardless of their individual or joint presence in the cell

**Fig. 1:**
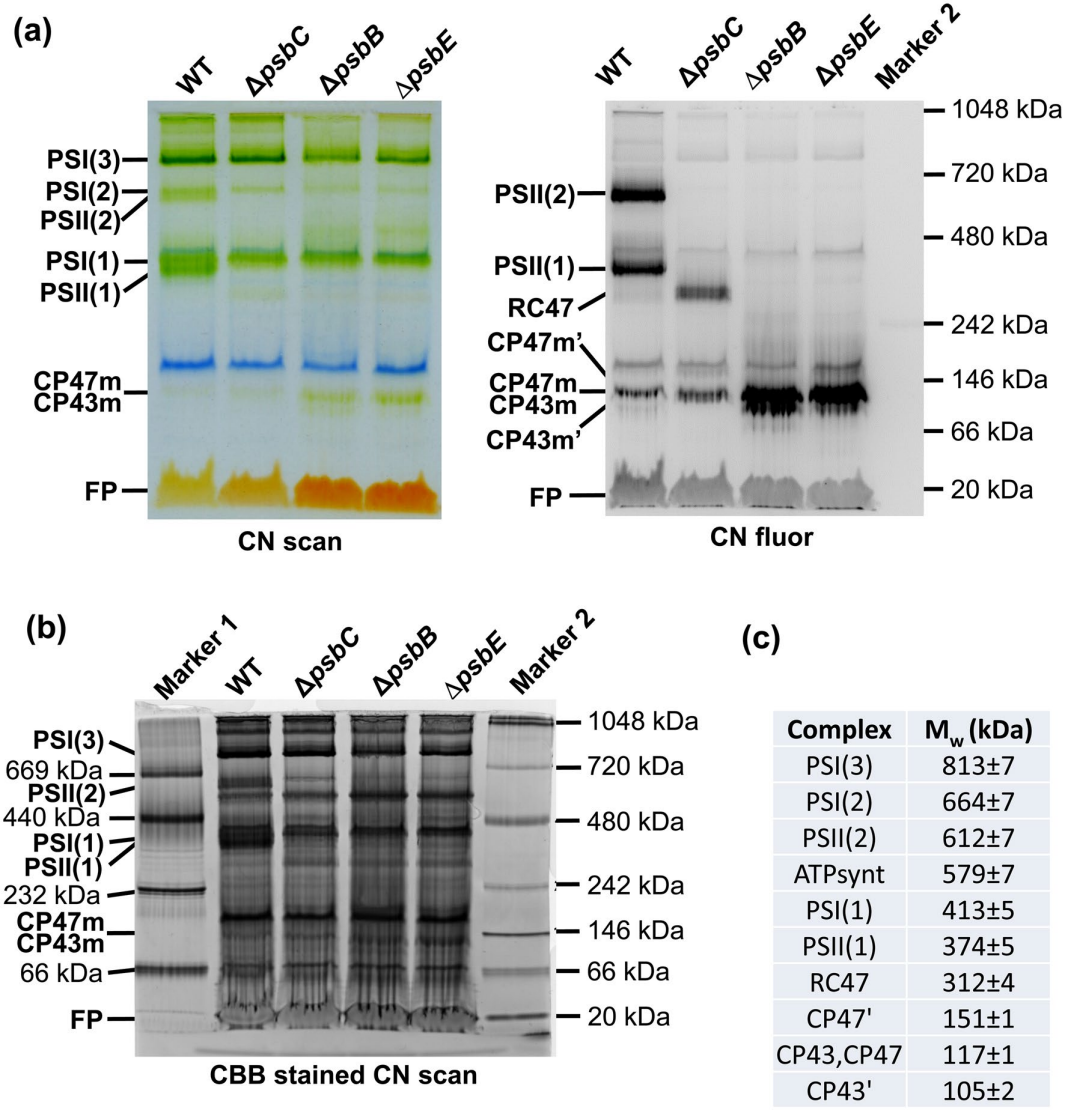
Analysis of membrane protein complexes of WT, Δ*psbC,* Δ*psbB and* Δ*psbE* strains using CN electrophoresis on 4–14% polyacrylamide gel. **a** Isolated membranes were solubilized with n-β-dodecyl-maltoside and after electrophoresis, the gel was scanned for colour (CN scan) and chlorophyll fluorescence (CN fluor). Designation of complexes: PSI(3) and PSI(1), trimeric and monomeric photosystem I; PSII(2) and PSII(1), dimeric and monomeric Photosystem II; RC47, monomeric PSII lacking CP43; u.CP47’ and u.CP47, unassembled CP47 with and without bound Psb35, respectively; u.CP43 and u.CP43’, CP43 with and without PsbZ; and FP, free pigments. The samples were loaded on the same OD_750nm_ basis corresponding to 4 µg of chlorophyll. **b** To visualize the markers, the gel in (**a**) was stained with Coomassie Brilliant Blue G-250 and scanned (CBB stained CN scan). Marker 1, high molecular weight calibration kit for electrophoresis (Amersham Biosciences); Marker 2, NativeMARK unstained protein standard (ThermoFisher). **c** The observed mass of protein complexes in gel (**b**) was calculated based on mobility of markers with known molecular mass

To address a possible formation of NRC, we first compared the electrophoretic mobility of free CP47m and CP43m, present, either together or separately, in *Synechocystis* cells. We analysed WT together with PSII mutants that are unable to synthesize CP43 (Δ*psbC*), CP47 (Δ*psbB*) or D2 due to the deletion of the *psbEFLJ* operon (Δ*psbE*) (Komenda et al. 2004). Solubilized membrane complexes of these strains were separated using a high-resolution CN PAGE. As expected, the fluorescing dimeric and monomeric PSII complexes [PSII(2) and PSII(1), resp.] were detectable only in WT (Fig. 1) while the trimeric and monomeric PSI complexes [PSI(3) and PSI(1), resp.] were present also in PSII mutants. The Δ*psbC* strain also contained the PSII core complex lacking CP43 (RC47) and in all mutants, the unassembled fluorescing antenna modules were migrating above free pigments (Fig. 1a). After Coomassie Blue staining of the CN gel (Fig. 1b), the mobilities of the main resolved complexes including the free modules were compared with mobilities of parallelly run standard marker proteins. The predicted molecular weights of CP47m and CP43m are 90 kDa and 81 kDa, respectively, and were calculated from the polypeptides and pigments comprising each module (Boehm et al. 2011; Komenda et al. 2012a): CP47m contains CP47 (55.9 kDa), PsbH (7.0 kDa), PsbL (4.3 kDa), PsbT (3.5 kDa), 16 chlorophylls and 3 β-carotenes, while CP43m contains CP43 (50.3 kDa), PsbK (5.1 kDa), PsbZ (6.7 kDa) and Psb30/Ycf12 (4.1 kDa), 13 chlorophylls and 4 β-carotenes. According to calibration curves constructed using protein standards, the relative molecular weight (M_W_) of the major CP47m and CP43m bands was around 120 kDa (Fig. 1c), which exceeds the theoretical values by about 50%.

**Fig. 2:**
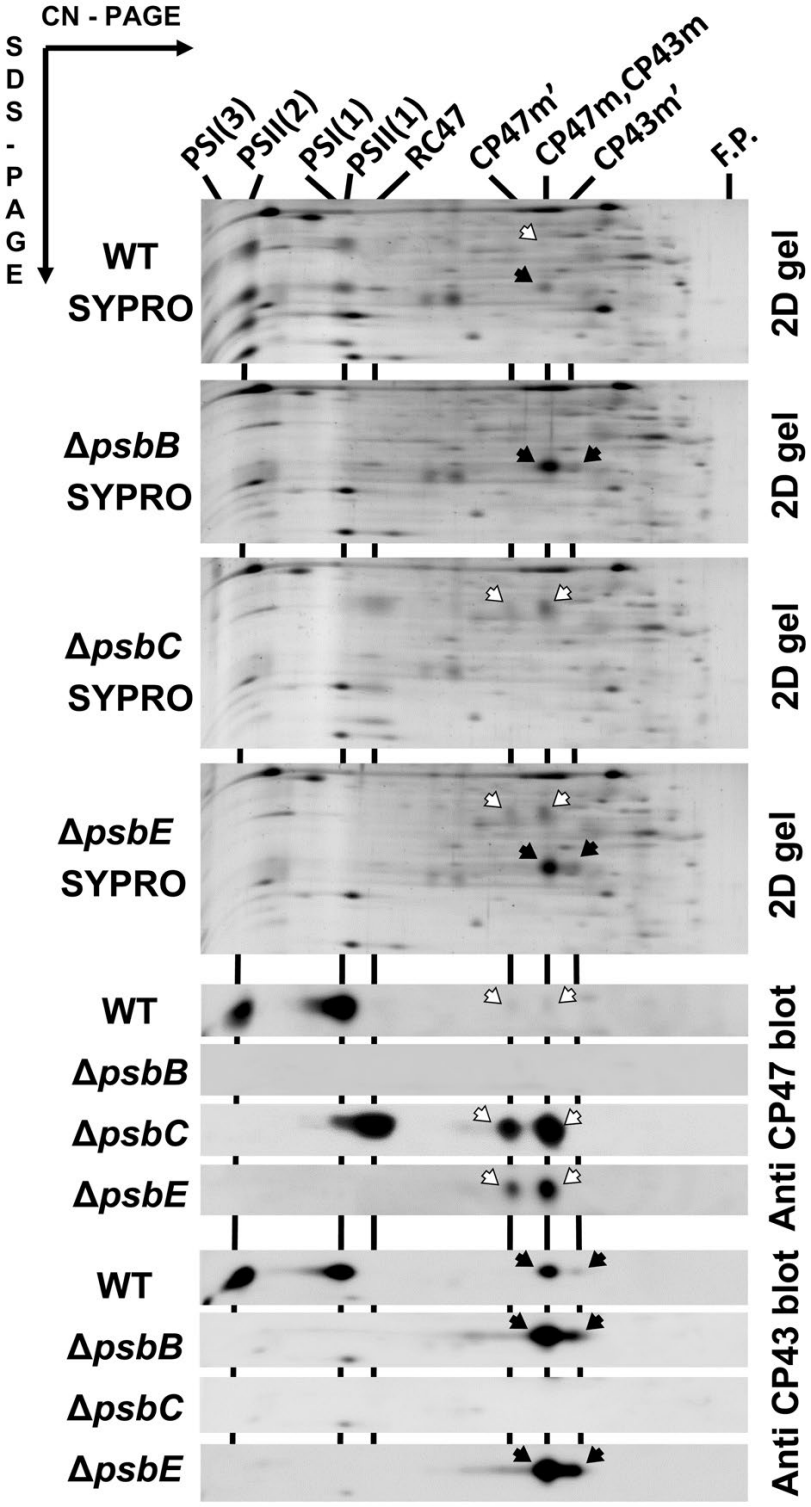
Analysis of membrane proteins of WT, Δ*psbC*, Δ*psbB* and Δ*psbE* strains using 2D CN/SDS PAGE. Membrane complexes separated as shown in Fig. 1 were analysed using SDS PAGE in the second dimension and after analysis, the gels were stained with SYPRO Orange (SYPRO), electroblotted and CP43 and CP47 proteins were detected using specific antibodies. Designation of complexes as in Fig. 1, RC47 is monomeric PSII lacking CP43. Black and empty arrows designate CP43 and CP47 spots in the 2D-stained gels and blots, respectively. Loading was as in Fig. 1

After analysis of individual complexes in all studied strains in the second dimension and precise alignment of the stained and immunodetected bands (Fig. 2), it became evident that the unassembled CP43m and CP47m were not homogeneous. Two minor module forms were found besides the major CP43m and CP47m bands with nearly identical mobilities. A faster migrating CP43m’ of about 105 kDa is known to lack at least the small subunit PsbZ (Komenda et al. 2012a) while a slower migrating CP47m’ (~ 150 kDa) contains an additional PSII assembly factor Psb35 (see Pascual-Aznar et al. 2021). The 2D gel analysis also proved the identity of larger fluorescing bands as PSII(2), PSII(1) and RC47 since they all contained CP47 and the first two also CP43. Most importantly, the main CP43 and CP47 modules had always identical electrophoretic mobilities (and therefore the same M_W_ of about 120 kDa) regardless of their occurrence individually (in Δ*psbC* and Δ*psbB*) or together with the second antenna module (in WT and Δ*psbE*). Thus, the formation of putative NRC was not observed in any of the *Synechocystis* PSII mutants assessed in this study*.*

**Fig. 3:**
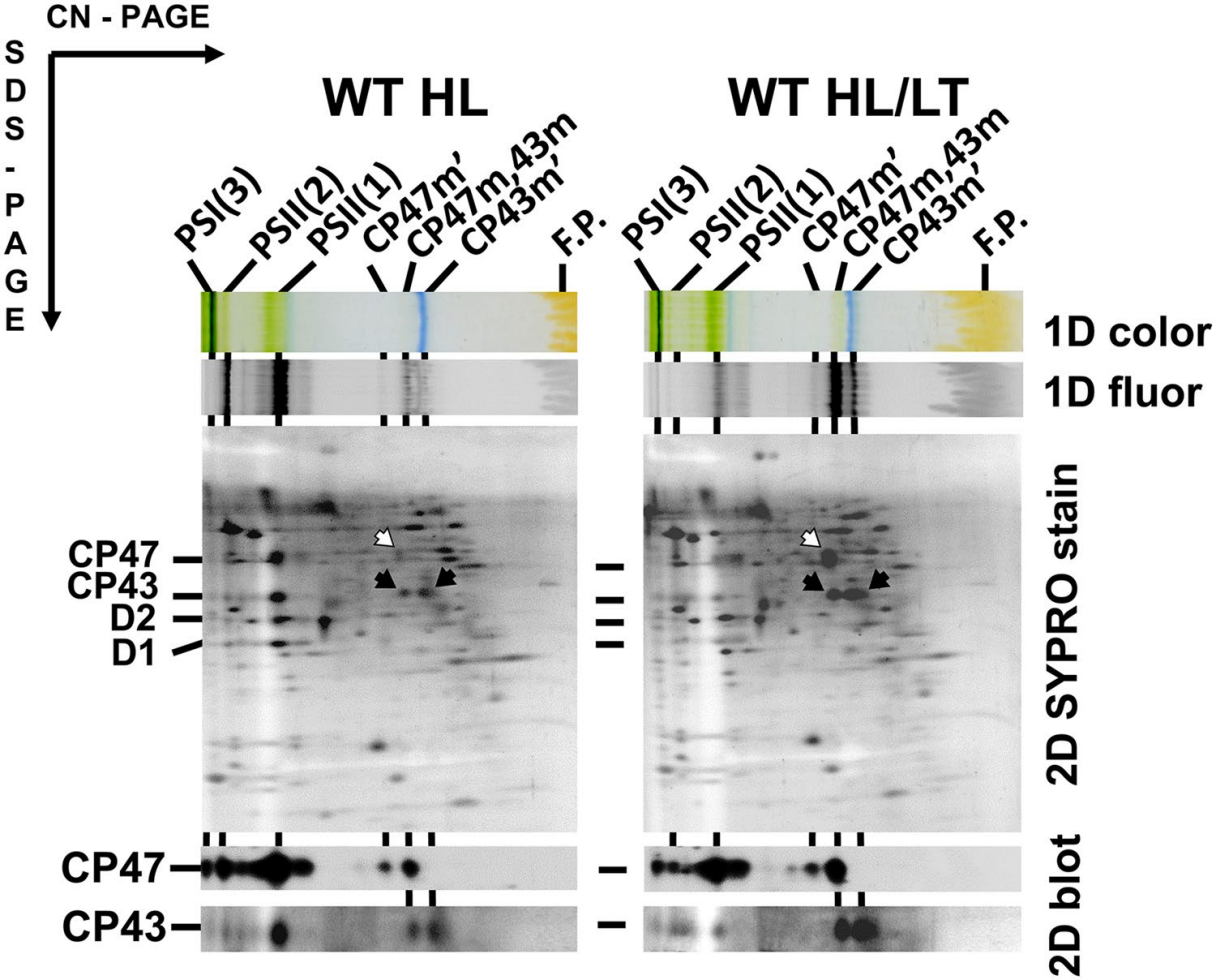
Analysis of membrane proteins of WT treated with high light and cold stress. Cells of WT were treated with high light (300 µmol photons m^−2^ s^−1^) for 60 min at 29 °C (WT HL) or with high light (300 µmol photons m^−2^ s^−1^) for 60 min at 18 °C (WT HL/LT). Membranes isolated from these cells were solubilized with n-β-dodecyl-maltoside and after analysis on 6–14% polyacrylamide, CN gel was scanned for colour (1D colour) and chlorophyll fluorescence (1D fluor). Membrane complexes were then analysed using SDS PAGE in the second dimension and after analysis, the gel was stained with SYPRO Orange (2D SYPRO stain), electroblotted and CP47 and CP43 were detected using specific antibodies (2D blot). Designation of complexes and proteins as in Figs. 1 and 2. 4 µg of chlorophyll was loaded for each membrane preparation

We further tested the proposed formation of a putative NRC complex in WT cells after degradation of D1 and D2 proteins. We analysed membranes isolated from WT cells exposed to either high light (HL) or to a combination of HL and low temperature (LT), which inhibits PSII repair. Indeed, the 2D CN/SDS PAGE revealed an increased accumulation of unassembled CP43 and CP47 modules under stress conditions in comparison with the control WT cells due to the disassembly of damaged PSII core complexes (Fig. 3). The 2D SYPRO-stained gels and immunodetection confirmed the presence of the major CP47m and small amount of CP47m’ like in the Δ*psbC* mutant while there was a higher ratio of CP43m’/CP43m than in the Δ*psbB* and Δ*psbE* strains. Mobility of all observed modules was the same as those observed in the control WT and PSII mutant cells and, once again, there was no indication of a larger CP43m-CP47m (NRC) complex.

### The isolated CP47 and CP43 modules do not co-purify with each other

**Fig. 4:**
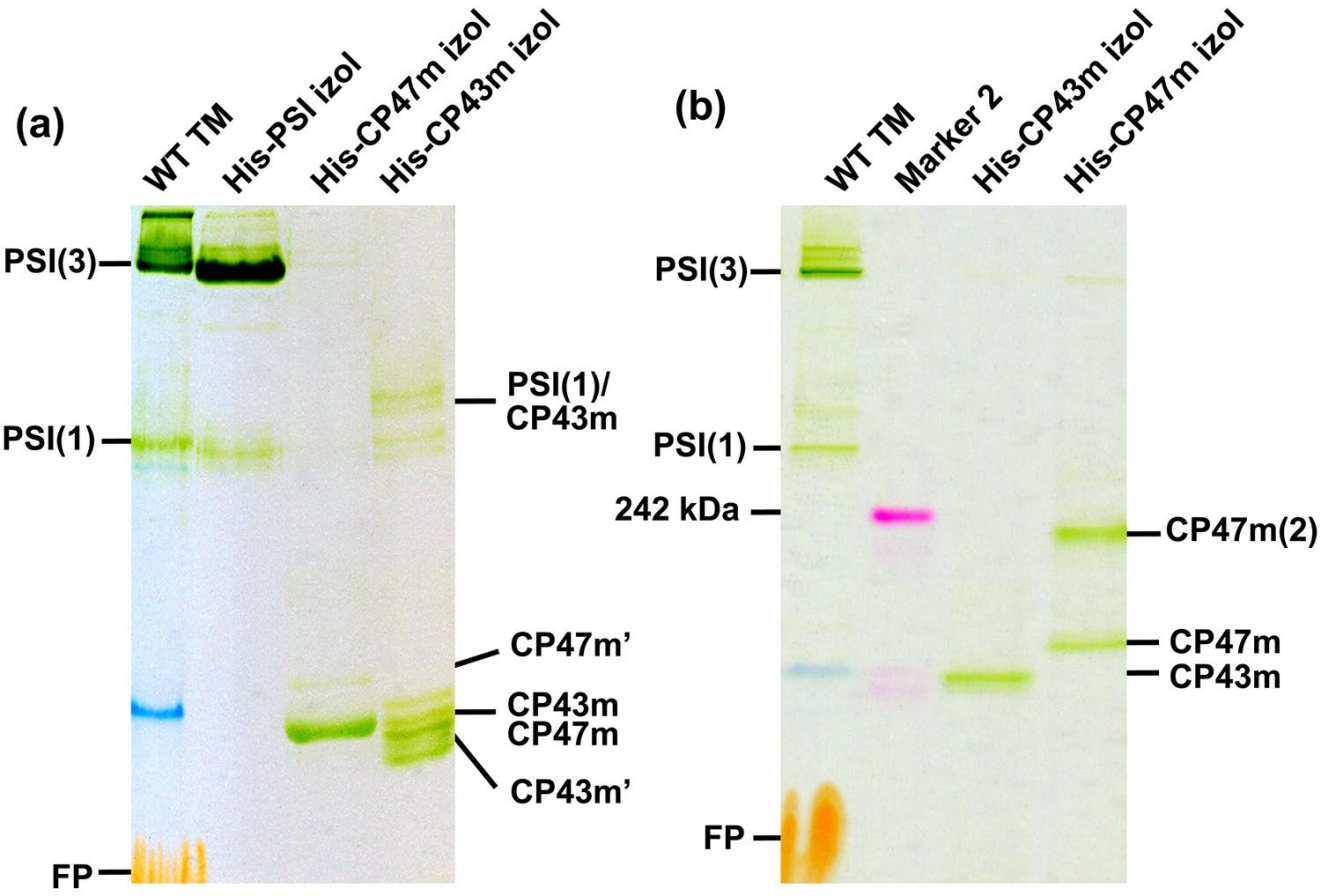
Analysis of purified His-tagged CP43 and CP47 assembly modules using CN native gels. **a** His-PSI, His-CP47 and His-CP43 complexes were purified using nickel affinity chromatography from *his-psaF*, *his-psbH*/Δ*psbH*/Δ*psbE*/Δ*ftsH2* and *his-psbC/*Δ*psbA* mutant strains. Isolated proteins were separated on 4–14% clear native gel (Komenda 2012a, b) together with WT membranes solubilized with 1% n-β-dodecyl-maltoside (w/v). Designation of complexes as in Figs. 1, 2 and 3, PSI(1)/CP43m is the monomeric photosystem I with bound CP43m; **b** His-CP43 and His-CP47 modules were purified from *his-psbC*/Δ*psbA* and *his-psbH*/Δ*psbH*/Δ*psbE*/Δ*ftsH2*-mutant strains, respectively, and separated on the 4–16% NativePAGE gel (ThermoFisher) (~ 0.5 mg of chlorophyll for the sample) together with WT membranes solubilized with 1% n-β-dodecyl-maltoside (w/v). Designation of complexes as in (**a**), CP47(2) refers to a dimer of CP47 module

To further clarify the possible formation of NRC, we isolated His-tagged CP47m and CP43m complexes from the strains lacking D1 or D2 proteins and therefore containing both assembly modules (Komenda et al. 2012a, b). The modules were initially isolated using the MES buffer of pH 6.5 and analysed using our standard gel system (Komenda et al. 2012a), which is slightly modified from (Wittig et al. 2007) having pH of gel buffer about 7 (Fig. 4a). The His-CP47 preparation contained the main band of CP47m and a small amount of a slower migrating CP47m’ as detected in membranes of the Δ*psbE* strain (Fig. 2). The preparation of His-CP43 showed a higher heterogeneity containing not only the main CP43m band but also smaller bands related to a partial cleavage of the lumenal loop of CP43 and loss of small subunits PsbZ and PsbK (Komenda et al. 2012a). In addition, a small amount of free or CP43m-associated PSI monomer was also present in the preparation as judged from the comparison with the isolated PSI using His-tagged PsaF. Nevertheless, the mobilities of both main CP47m and CP43m modules were again similar and, importantly, each module was completely free of the other module.

To exclude that the hypothetical interaction between CP47m and CP43m is specifically disrupted in low pH, we isolated both His-tagged modules also in HEPES buffer B of pH 7.5 and analysed them using the NativePAGE precast gel with buffer pH of about 7.5 (Fig. 4b). Again, both modules contained no admixture of the other module. Surprisingly, the His-CP47m preparation separated on NativePAGE migrated in two bands, one corresponding to CP47m of about 120 kDa and the other corresponding most probably to a dimer of CP47m with the approximately twofold M_W_. In contrast, His-CP43m was very homogenous indicating higher stability of this module under higher pH.

## Discussion

The CP47 and CP43 antennas together with their adjacent subunits were recently proposed to form NRC, a complex that putatively improves the efficiency of the PSII repair cycle by preventing NRC components from unnecessary damage and degradation (Weisz et al. 2019). After years of intensive study of the PSII life cycle and photoinhibition (for review see Adir et al. 2003; Theis and Schroda 2016), the discovery of such a complex was surprising because it had never been discussed in earlier literature. Indeed, how CP47m and CP43m could bind in a stable complex is difficult to imagine because they localize at the opposite ends of the D1-D2 heterodimer and are rather far away (Online Resource 1). We note that published evidence for NRC presence (Weisz et al. 2019) lacks a control mobility experiment with individually isolated CP47m or CP43m. It is well known that the mobility for membrane proteins is different from that predicted from amino acid composition; typically, it is much lower (Strecker et al. 2010). The reasons for lower mobility of membrane proteins are their irregular, non-spherical shape and especially binding of detergents and lipids that form a shell around the protein (Strecker et al. 2010). Therefore, in the present study, we compared CP47m and CP43m mobilities in the WT and PSII mutants which accumulate CP47m, CP43m or both modules together. The mobility was assessed using high-resolution and mild CN PAGE in the first dimension and SDS PAGE in the second dimension followed by immublotting. The results showed that the main forms of CP47m and CP43m have nearly identical mobilities, which do not allow their clear separation based on size only. Thus, almost the same mobility is seen for the individual CP43m in the strain lacking CP47 and, vice versa, for the individual CP47m in the absence of CP43. Moreover, identical mobilities were also observed for CP47m and CP43m in the stressed WT cells and in Δ*psbE* cells, in which both modules exist together due to the degradation of D1 and D2 and due to the absence of D2 accumulation (Komenda et al. 2004), respectively. Since these modules should be present in the form of NRC*,* the result strongly questions the existence of NRC.

Weisz et al. (2019) argued that NRC forms during PSII repair and not during the de novo PSII assembly. However, the experiment supporting this hypothesis is not convincing because the amount of newly synthesized CP47m and CP43m in WT cells is negligible and they cannot be distinguished from CP47m and CP43m released after degradation of D1 and D2 unless radioactive labelling is used. Weisz et al. (2019) also proposed the separation of RCII and NRC before the degradation of D1 and D2. However, such a proposal is at odds with previously published data. Firstly, the detachment of RCII and formation of NRC would necessarily lead to much faster D2 degradation since in the CP47-less strain accumulating RCII, the degradation of D2 is even faster than that of D1, most probably due to the accessibility of the N-terminus of D2 to the FtsH protease (Krynická et al. 2015). Quantitative protein labelling data document that D1 is selectively turned over in vivo and has a lifetime ten times shorter than the lifetime of D2 (Yao et al. 2012). This selectivity agrees with the specific detachment of CP43m, which is bound at the D1 side of PSII much weaker than CP47 at the D2 side (Zheleva et al. 1998). This detachment results in the well-documented transient formation of RC47 (Adir et al. 1990; Barbato et al. 1992; Komenda and Masojídek 1995), which also accumulates when D1 cannot be degraded either due to the absence of the FtsH protease (Komenda et al. 2006) or due to the N-terminal truncation of D1 (Komenda et al. 2007). Thus, we consider the RCII – NRC separation quite improbable although when new D1 synthesis is stopped, the D1 degradation is slowed down and D2 might be degraded at a rate more resembling that of D1 (Komenda and Barber 1995; Komenda and Masojídek 1995).

Our estimation of M_W_ of each module (120 kDa) was based on comparison with mobility of soluble marker proteins during CN PAGE and it is about 30–40 kDa higher than the theoretical M_W_ (Boehm et al. 2011). Since pI of CP47 and CP43 are not anomalous (5.5 and 6.1, respectively), this difference can be mainly ascribed to the presence of lipidic/detergent shell around the membrane apoproteins (Strecker et al. 2010). Zouni et al. (2005) estimated that the shell of CP47 is about 50 kDa, which is in a reasonable agreement with its M_W_ determined by us if we account for larger lipidic content due to the presence of additional small protein subunits. CN PAGE also allowed separation of minor module forms CP47’ and CP43’; see Fig. 2 and the SYPRO Orange-stained 2D gel of Δ*psbE* (Fig. 3). As described earlier, these forms differ from the standard ones by the presence (for CP47’m) or absence (for CP43’m) of the small membrane subunits Psb35 and PsbK/Z, respectively. However, the changed mobility of these minor modules corresponded to much larger gain/loss in Mw calculated from the electrophoretic mobility of soluble proteins (Fig. 1c). Thus, the results showed the high resolving power of our CN PAGE approach, which is sufficient for clear separation of membrane complexes differing in a single small 10 kDa membrane protein. Distinguishing individual holo-modules of 80–90 kDa and their mutual complex of the twofold size by CN PAGE must therefore be unequivocal. If CP47m and CP43m made a NRC complex, its mobility would have been close to the mobility of dimeric CP47m observed in the preparation of the isolated His-tagged CP47m analysed in the precast CN gel (Fig. 4), but an additional band of such size containing both CP47 and CP43 was observed neither in Δ*psbE* nor in stressed WT cells (Figs. 2 and 3).

The resolving power of gradient sucrose or glycerol ultracentrifugation is weaker in comparison with CN PAGE; even PSII(1) and RC47 differing by the presence of CP43m can hardly be separated using this method (Zhang et al. 1999; Laczkó-Dobos et al. 2008). Therefore, a subtle difference between the mobility of both modules cannot be detected. M_W_ of the putative NRC (170 kDa) was determined by following its sedimentation in the ultracentrifugal field and subsequent calculation assuming the standard behaviour of the complex as a spherical particle of a specific volume (Zouni et al. 2005; Weisz et al. 2019). This value differs even more from the theoretical value predicted just from the amino acid sequence and known pigments composition than the one derived from CN PAGE. We think this is again due to the lipidic and detergent shell, which might be preserved even more than during CN PAGE, and also because membrane-bound modules in reality are not an ideal spherical particles. The putative NRC complex was mainly isolated from the strain expressing His-tagged CP47 and lacking PsbO (Weisz et al. 2019), which has been previously shown to suffer from light-induced damage and fast D1 and D2 degradation (Komenda and Barber 1995). This PsbO-less strain therefore accumulates an increased level of CP43m and especially CP47m in comparison with WT (Komenda et al. 2010). Consequently, the use of nickel affinity chromatography for the purification of His-CP47 complexes results most probably in isolating a mixture of His-CP47m, His-RC47 and His-PSII(1). The subsequent analysis of the preparation using CN PAGE or glycerol density centrifugation also resulted in partial detachment of loosely bound CP43m from PSII(1) (see, for instance Kiss et al. 2019) and the putative NRC complex in fact consists of a mixture of isolated His-CP47m and CP43m detached from the isolated His-PSII(1).

We also isolated the His-tagged CP47m and CP43m and under our standard CN gel electrophoresis conditions, they migrated near identically to each other. Each module was absolutely free of the other despite their joint occurrence in the D1- and D2-less cells used for isolation (see also Boehm et al. 2011). Interestingly, when isolated and analysed at higher pH, CP47m had a tendency to aggregate forming the molecular species of about 230 kDa corresponding well to a dimer. Indeed, such size should be expected for the putative CP47m-CP43m complex separated in our standard CN gel. We note that the observed CP47m dimer is most likely an artefact of native electrophoresis because only a monomeric CP47m was detected using size exclusion chromatography of the His-CP47 preparation in the same HEPES buffer (D’Haene et al. 2015).

## Conclusion

In conclusion, we show that CP47m and CP43m in *Synechocystis* have very similar mobilities when separated using CN PAGE regardless of whether they are present in the cells alone or together. Their M_W_ derived from comparison of their mobility with soluble marker proteins of known composition is significantly larger than M_W_ calculated from the protein subunits and pigments present in the modules but it does not reach the value expected for the proposed NRC complex. Moreover, His-tagged modules do not co-isolate with complementary antenna modules isolated from strains lacking D1 or D2. The presented data do not confirm the formation of the stable NRC complex in the absence of D1 and D2 and, therefore, we conclude that NRC most likely represents a mixture of unassembled antenna modules whose larger mass, as previously determined by analytical centrifugation, comes from their lipidic/detergent shell and irregular shape.

## Supplementary Information

Below is the link to the electronic supplementary material.

Supplementary file1 (PDF 821 kb)

## References

[CR1] Adir N, Shochat S, Ohad I (1990). Light-dependent D1 protein synthesis and translocation is regulated by reaction center II. Reaction center II serves as an acceptor for the D1 precursor. J Biol Chem.

[CR2] Adir N, Zer H, Shochat S (2003). Photoinhibition – a historical perspective. Photosynth Res.

[CR3] Barbato R, Friso R, Rigoni F (1992). Structural changes and lateral redistribution of photosystem II during donor side photoinhibition of thylakoids. J Cell Biol.

[CR4] Barber J (1995). Molecular Basis of the Vulnerability of Photosystem II to Damage by Light. Austr J Plant Physiol.

[CR5] Boehm M, Romero E, Reisinger V (2011). Investigating the early stages of photosystem II assembly in Synechocystis sp. PCC 6803 isolation of CP47 and CP43 complexes. J Biol Chem.

[CR6] D’Haene S, Sobotka R, Bučinská L (2015). Interaction of the PsbH subunit with a chlorophyll bound to histidine 114 of CP47 is responsible for the red 77 K fluorescence of Photosystem II. Biochim Biophys Acta Bioenerg.

[CR7] Eaton-Rye JJ, Vermaas WFJ (1991). Oligonucleotide-directed mutagenesis of psbB, the gene encoding CP47, employing a deletion mutant strain of the cyanobacterium Synechocystis sp. PCC 6803. Plant Mol Biol.

[CR8] Kiss E, Knoppová J, Pascual Aznar G (2019). A photosynthesis-specific rubredoxin-like protein is required for efficient association of the D1 and D2 proteins during the initial steps of photosystem II assembly. Plant Cell.

[CR9] Komenda J, Barber J (1995). Comparison of psbO and psbH deletion mutants of *Synechocystis* PCC 6803 indicates that degradation of D1 protein is regulated by the QB site and dependent on protein synthesis. Biochem.

[CR10] Komenda J, Masojídek J (1995). Structural changes of Photosystem II complex induced by high irradiance in cyanobacterial cells. Eur J Biochem.

[CR12] Komenda J, Reisinger V, Muller BC (2004). Accumulation of the D2 protein is a key regulatory step for assembly of the photosystem II reaction center complex in *Synechocystis* PCC 6803. J Biol Chem.

[CR13] Komenda J, Barker M, Kuviková S (2006). The FtsH protease Slr0228 is important for quality control of photosystem II in the thylakoid membrane of *Synechocystis* sp PCC 6803. J Biol Chem.

[CR14] Komenda J, Tichý M, Prášil O (2007). The exposed N-terminal tail of the D1 subunit is required for rapid D1 degradation during photosystem II repair in *Synechocystis* sp PCC 6803. Plant Cell.

[CR15] Komenda J, Knoppová J, Krynická, V (2010). Role of FtsH2 in the repair of photosystem II in mutants of the cyanobacterium Synechocystis PCC 6803 with impaired assembly or stability of the CaMn_4_ cluster. Biochim Biophys Acta Bioenerg.

[CR16] Komenda J, Knoppová J, Kopečná J (2012). The Psb27 assembly factor binds to the CP43 complex of photosystem II in the cyanobacterium Synechocystis sp PCC 6803. Plant Physiol.

[CR17] Komenda J, Sobotka R, Nixon PJ (2012). Assembling and maintaining the photosystem II complex in chloroplasts and cyano-bacteria. Cur Opin Plant Biol.

[CR18] Krynická V, Shao S, Nixon PJ (2015). Accessibility controls selective degradation of photosystem II subunits by FtsH protease. Nature Plants.

[CR19] Kubota H, Sakurai I, Katayama K (2010). Purification and characterization of photosystem I complex from Synechocystis sp PCC 6803 by expressing histidine-tagged subunits. Biochim Biophys Acta Bioenerg.

[CR20] Laczkó-Dobos H, Ughy B, Tóth SZ (2008). Role of phosphatidylglycerol in the function and assembly of photosystem II reaction center, studied in a cdsA-inactivated PAL mutant strain of *Synechocystis* sp. PCC6803 that lacks phycobilisomes. Biochim Biophys Acta Bioenerg.

[CR21] Pakrasi HB, Williams JG, Arntzen CJ (1988). Targeted mutagenesis of the *psbE* and *psbF* genes blocks photosynthetic electron transport: evidence for a functional role of cytochrome b559 in photosystem II. EMBO J.

[CR22] Pascual-Aznar G, Konert G, Bečková M (2021). Psb35 protein stabilizes the CP47 assembly module and associated high-light inducible proteins during the biogenesis of photosystem II in the cyanobacterium *Synechocystis* sp. PCC6803. Plant Cell Physiol.

[CR23] Shukla MK, Llansola-Portoles MJ, Tichý M (2018). Binding of pigments to the cyanobacterial high-light-inducible protein HliC. Photosynth Res.

[CR24] Silva P, Thompson E, Bailey S (2003). FtsH is involved in the early stages of repair of photosystem II in Synechocystis sp PCC 6803. Plant Cell.

[CR25] Strecker V, Wumaier Z, Wittig I (2010). Large pore gels to separate mega protein complexes larger than 10 MDa by blue native electrophoresis: isolation of putative respiratory strings or patches. Proteomics.

[CR26] Theis J, Schroda M (2016). Revisiting the photosystem II repair cycle. Plant Signal Behav.

[CR27] Tichý M, Bečková M, Kopečná J (2016). Strain of *Synechocystis* PCC 6803 with aberrant assembly of photosystem II contains tandem duplication of a large chromosomal region. Front Plant Sci.

[CR28] Vermaas WF, Ikeuchi M, Inoue Y (1988). Protein composition of the photosystem II core complex in genetically engineered mutants of the cyanobacterium *Synechocystis* sp. PCC 6803. Photosynth Res.

[CR29] Weisz DA, Johnson VM, Niedzwiedzki DM (2019). A novel chlorophyll protein complex in the repair cycle of photosystem II. Proc Natl Acad Sci USA.

[CR30] Wittig I, Karas M, Schagger H (2007). High resolution clear native electrophoresis for in-gel functional assays and fluorescence studies of membrane protein complexes. Mol Cell Proteomics.

[CR31] Yao DCI, Brune DC, Vermaas WFJ (2012). Lifetimes of photosystem I and II proteins in the cyanobacterium Synechocystis sp. PCC 6803. FEBS Lett.

[CR32] Zhang L, Paakkarinen V, van Wijk KJ (1999). Co-translational assembly of the D1 protein into photosystem II. J Biol Chem.

[CR33] Zheleva D, Sharma J, Panico M (1998). Isolation and characterization of monomeric and dimeric CP47-reaction center photosystem II complexes. J Biol Chem.

[CR34] Zouni A, Kern J, Frank J (2005). Size determination of cyanobacterial and higher plant Photosystem II by gel permeation chromatography, light scattering, and ultracentrifugation. Biochem.

