# Supplementary material for: Photosystem II antenna modules CP43 and CP47 do not form a stable ‘no reaction centre complex’ in the cyanobacterium Synechocystis sp. PCC 6803
Source: Photosynth Res. 2022 Jan 11;152(3):363–71. doi: 10.1007/s11120-022-00896-w (PMC9458580; doi:10.1007/s11120-022-00896-w)
Supplement: Supplementary file 1 — Supplementary file1 (PDF 821 kb) [file 11120_2022_896_MOESM1_ESM.pdf]

Photosynthesis Research

Photosystem II antenna modules CP43 and CP47 do not form a "no reaction centre complex" in the cyanobacterium *Synechocystis* sp. PCC 6803

Martina Bečková [1], Roman Sobotka[1] and Josef Komenda [1, *]

[1]Institute of Microbiology of the Czech Academy of Sciences, Centre Algatech, Laboratory of Photosynthesis, Opatovický mlýn, 37981 Třeboň, Czech Republic

*Correspondence: komenda@alga.cz; Tel.: (+420 384 240 431)

Online Resource

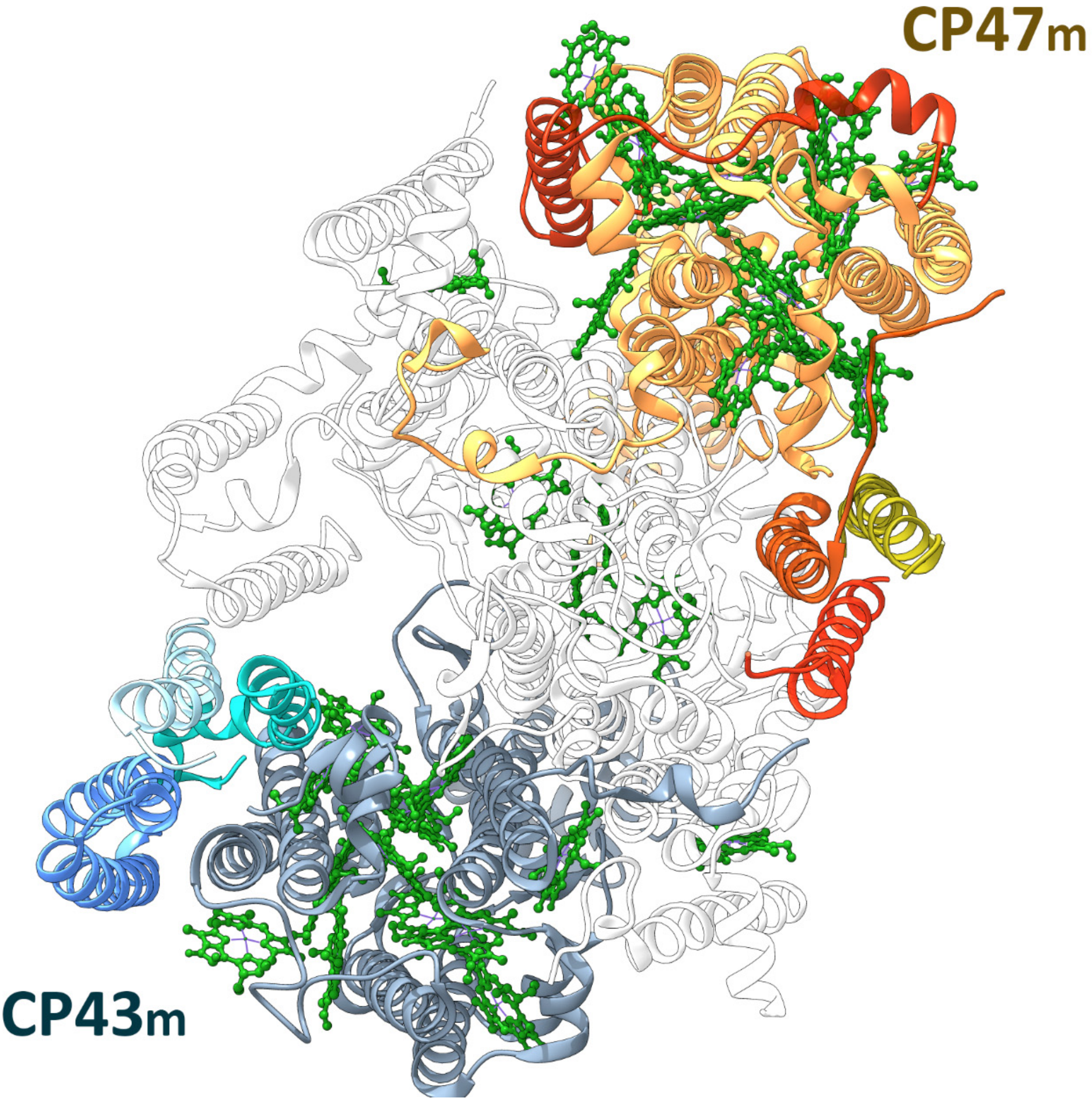

Online Resource 1. The cartoon of Photosystem II documenting the gap between CP47 and CP43 modules when the D1 and D2 modules are degraded. The cartoon is based on PSII model of Umena et al. 2011 (PDB code 3WU2). Color code: CP47 amber; PsbH dark red; PsbL, orange; PsbM, light red; PsbT yellow-green; CP43, grey; PsbK, turquoise; PsbZ, dark blue; Psb30/Ycf12, light blue; Chls, green. Contours of D1, D2 and the adjacent small subunits PsbE, PsbF, PsbI and PsbX are just outlined.
